# Magnesium and Zinc Are Associated with Sleep Quality in Saudi Adults: Evidence from a Cross-Sectional Study

**DOI:** 10.3390/nu18010114

**Published:** 2025-12-29

**Authors:** Sara Al-Musharaf, Madhawi M. Aldhwayan, Tagreed A. Mazi, Ohud Abujabir, Waad Alfawaz, Ghadeer S. Aljuraiban

**Affiliations:** 1 Community Health Sciences Department, College of Applied Medical Sciences, King Saud University, Riyadh P.O. Box 10219, Saudi Arabia; maldhwayan@ksu.edu.sa (M.M.A.); tmazi@ksu.edu.sa (T.A.M.); walfawaz@ksu.edu.sa (W.A.); galjuraiban@ksu.edu.sa (G.S.A.); 2 Center of Excellence in Biotechnology Research (CEBR), King Saud University, Riyadh 11451, Saudi Arabia

**Keywords:** zinc, magnesium, copper, Pittsburgh Sleep Quality Index, Middle East and North Africa

## Abstract

Background: Studies that examine magnesium (Mg), zinc (Zn), and copper (Cu) in relation to sleep in the Middle East and North Africa are limited. We aim to assess the associations between serum and dietary Mg, Zn, and Cu levels and sleep quality in Saudi adults. Methods: A cross-sectional study was conducted among 1041 adults. Sleep quality was assessed using the Arabic Pittsburgh Sleep Quality Index (PSQI). Serum mineral levels were quantified biochemically, and dietary intake information was obtained. We utilized logistic regression to estimate the odds ratios for poor sleep (PSQI-P) in relation to serum and dietary indices in a crude model and after adjustment for confounders. Results: Serum Mg deficiency (<1.8 mg/dL) increased the odds of PSQI-P by 30% in the crude and age-adjusted models, with attenuation after further adjustment, suggesting partial mediation by lifestyle and metabolic factors. Mg deficiency was associated with PSQI-P (1.8-fold higher odds) after full adjustment. Dietary Mg levels below the DRI were independently associated with poor sleep across models. Higher serum Zn tertiles were associated with 40% lower odds of PSQI-P, and Zn deficiency (<80 µg/dL) demonstrated a three-fold independent increase in risk. Neither serum nor dietary intake levels of Cu demonstrated an association with sleep quality. Conclusion: In Saudi adults, serum and dietary Mg levels were associated with poor sleep, particularly in males, while the serum Zn concentration exhibited a modest inverse association at higher levels. Further longitudinal studies are warranted.

## 1. Introduction

Sleep is a vital biological process essential for cognitive function, emotional regulation, and the maintenance of overall physiological health [1]. Current guidelines recommend that adults maintain 7–9 h of sleep per night to support optimal health and daytime performance [2,3]. However, suboptimal sleep quality has represented a prevalent global challenge from 2010 to 2024, affecting approximately 40% of the global adult population [4], with disparities reported across ages, sexes, occupations, and geographic regions [5,6,7,8]. In Saudi Arabia, data indicate a concerning 25–80% prevalence of poor sleep quality across various adult populations [9,10,11,12], highlighting poor sleep as a substantial public health issue. Epidemiological data indicate that suboptimal sleep is associated with adverse health outcomes, including increased risks of mortality, cardiovascular disease, diabetes, cancer, and mental health conditions [13,14,15,16,17,18].

Several modifiable lifestyle, psychological, and environmental factors are thought to contribute to poor sleep quality [19,20,21,22,23]. In addition, a growing body of evidence highlights the potential contribution of micronutrient(s), including the major mineral magnesium (Mg) and the trace elements zinc (Zn) and copper (Cu), in modulating sleep quality. The mechanism by which these minerals affect sleep physiology is not fully understood; however, it involves a role of these minerals in the synthesis, transport, and receptor modulation of key neurotransmitters, such as γ-aminobutyric acid (GABA), the glutamate N-methyl-D-aspartate (NMDA) receptor, and dopamine, and the regulation of sleep-promoting hormones, including serotonin and melatonin [24,25,26].

Multiple studies have demonstrated an inverse association between serum/plasma Zn and Mg and the parameters determining poor sleep quality [24,27,28,29,30,31,32,33]. The findings for Cu are inconsistent, with some studies reporting a negative association with sleep quality [30,34], whereas others report no association [28]. Another study reported a U-shaped association [35]. Additionally, several studies reported that a lower serum Cu/Zn ratio (or a higher Zn/Cu ratio) is associated with better sleep outcomes [28,29,30,34]. However, most of the available studies examining the association between Mg, Zn, and Cu and sleep quality are predominantly from Western populations and often use unvalidated self-reported measures for sleep duration, quality, and disorders [28,30,36].

Many prior investigations were restricted to sex-specific or high-risk groups, limiting their generalizability to the broader population [31,34,37,38], and none examined both serum and dietary levels simultaneously in the same population. Importantly, studies originating from the Middle East and North Africa (MENA) region are limited by small sample sizes and highly selective populations [27,39,40,41]. To address this gap, we examined the associations between serum and dietary Mg, Zn, and Cu levels and sleep quality, assessed using the Pittsburgh Sleep Quality Index (PSQI), in a cohort of Saudi adults. We also evaluated combined serum mineral indices and the Cu/Zn ratio. We hypothesized that lower serum and dietary levels of Mg, Zn, and Cu and a higher Cu/Zn ratio would be associated with poorer sleep quality, reflected by higher PSQI scores.

## 2. Materials and Methods

### 2.1. Study Design and Settings

In this cross-sectional study, data were collected between December 2021 and December 2023. The study protocol was approved by the Research Ethics Committee and Institutional Review Board at the College of Medicine, King Saud University, Riyadh, Saudi Arabia (KSU-IRB-21-314, 25 March 2022). Before enrollment, study participants provided consent following an explanation of the study protocol. Participant confidentiality was maintained through encrypted data storage systems and anonymized analytical procedures.

### 2.2. Study Population

The study subjects were a sample of apparently healthy adults recruited via advertisements on social media platforms and from two commercial shopping centers in Riyadh, Saudi Arabia (Al-Nakheel Mall and Al-Hamra Mall). The inclusion criteria included male and female Saudi adults, aged ≥18 years, residing in Riyadh, and capable of providing informed consent. The exclusion criteria included pregnant or lactating women, a history of alcohol consumption, the use of medications known to influence Mg, Cu, or Zn metabolism, and the presence of a chronic disease, including type 2 diabetes, chronic kidney or liver disease, cancer, Crohn’s disease, or ulcerative colitis, as reported by participants.

### 2.3. Sample Size and Sampling Method

**Figure 1:**
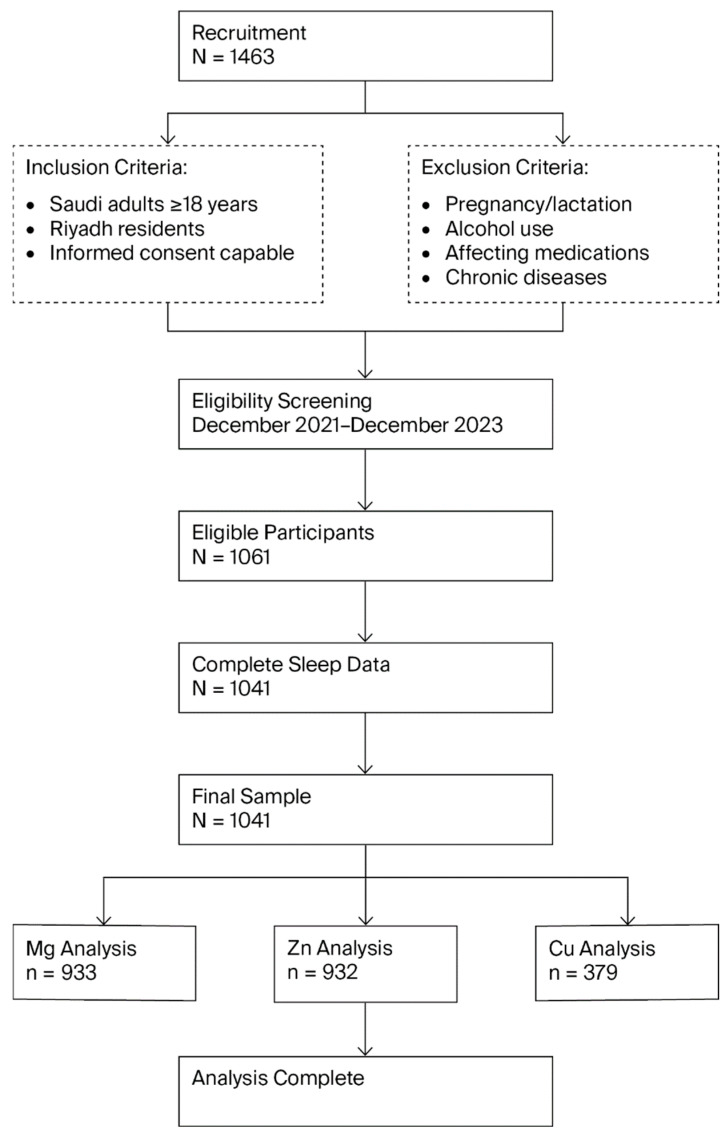
Flow chart of the study. Cu, Copper; Mg, Magnesium; and Zn, Zinc.

Sample size determination was based on the associations between serum Zn levels and sleep quality reported by Ji and colleagues [33], who identified an OR of 1.58 for compromised sleep among individuals with low serum Zn levels. Utilizing G*Power statistical software (version 3.1; Heinrich-Heine-Universität Düsseldorf, Düsseldorf, Germany; http://www.gpower.hhu.de/ accessed 15 October 2023) [42], we performed a logistic regression analysis with the following parameters: OR = 1.58, 95% confidence interval (α = 0.05), and 80% statistical power (β = 0.2). This calculation yielded a minimum necessary sample of 172 participants per comparative group. Ultimately, 1041 participants with complete sleep assessment data were included. Serum samples were available for Mg (*n* = 933), Zn (*n* = 932), and Cu (*n* = 379) analyses (Figure 1).

### 2.4. Data Collection

At recruitment sites, participants were guided through a mobile research facility with multiple assessment stations. In the welcoming area, participants received an overview of the study’s objectives and protocol before consenting and progressing to the interview and anthropometric measurement stations. Before departure, participants were scheduled for a blood collection appointment within the next 14 days and given instructions to arrive fasted for blood collection.

#### 2.4.1. Socio-Demographics and Anthropometric Measurements

During the main visit, a trained dietitian administered questionnaires to collect information on socioeconomic status, including age and sex. In addition, anthropometric measurements were collected. Body weight was measured in kilograms using Bioelectrical Impedance Analysis (BIA) (InBody 570; Seoul, Republic of Korea), while height assessments were performed using a digital stadiometer (Seca 274; Hamburg, Germany). Measurements were performed without heavy clothing and shoes, recorded to the nearest 0.1 cm, and repeated to obtain mean values for subsequent analysis. Body mass index (BMI) was calculated following standard formulas (weight divided by height squared), expressed as kilograms per square meter (kg/m^2^), and classified according to established Centers for Disease Control categories [43]. For the body composition analysis, particularly fat percentage, we employed InBody 570 composition analyzers (InBody 570; Seoul, South Korea).

#### 2.4.2. Biochemical Blood Analysis

A certified phlebotomist collected venous blood samples following 8–10 h of fasting at Al-Farabi Medical Labs in Riyadh. Serum Mg was measured using a spectrophotometric methodology (Diatron P500 analyzer; Budapest, Hungary). Serum Cu and Zn determinations employed inductively coupled plasma dynamic reaction cell mass spectrometry, as described elsewhere. Mineral deficiency was determined based on established median thresholds for serum Mg (<1.8 mg/dL) [44], Cu (<119 μg/dL), and Zn (<80 μg/dL) [45]. The serum Cu/Zn ratio was calculated by dividing the serum Cu concentration by the serum Zn concentration. The Cu/Zn ratio was established at (>1.72) [46]. Lab results were shared with participants upon request.

#### 2.4.3. Sleep Quality Assessment

A validated Arabic version of the Pittsburgh Sleep Quality Index (PSQI) [47] was administered during the main visit to evaluate participants’ sleep characteristics. This instrument comprises 19 items organized across seven component domains: subjective sleep quality perception, sleep onset latency, sleep duration, habitual sleep efficiency, sleep disturbances, sleep medication utilization (both prescription and non-prescription), and daytime functional impairment. Each component is scored on a 0–3 scale (0 = not experienced during the past month, 1 = less than once weekly, 2 = once or twice weekly, 3 = three or more times weekly), yielding a cumulative score ranging from 0 to 21. Based on this score, participants with PSQI > 5 were categorized as having poor sleep quality (PSQI-P), while those with PSQI ≤ 5 were categorized as having good sleep quality (PSQI-G).

#### 2.4.4. Dietary Assessment

Dietary intake was assessed using three nonconsecutive 24 h dietary recalls (two weekdays and one weekend day) collected by a trained clinical dietitian. The first two recalls were collected during the main visit, and the third was collected over a phone interview administered within a week after the main visit. To improve data quality, all interviews were conducted by well-trained interviewers following a standardized protocol. The use of repeated recalls was intended to reduce random errors related to day-to-day variation in dietary intake. Recalls were analyzed with ESHA Food Processor SQL software (version 16.0; www.esha.com) to quantify macronutrient and micronutrient intakes, including Mg, Zn, and Cu. To enable comparisons independent of total energy intake, the mineral intake was energy-adjusted using a density approach and expressed per 1000 kcal. Dietary mineral intakes were further evaluated against sex-specific Dietary Reference Intakes (DRIs), with suboptimal intake defined as Mg < 420 mg/day for males and <320 mg/day for females, Zn < 11 mg/day for males and <9 mg/day for females, and Cu < 900 mg/day for males and females [48], and these cut-offs were used to classify participants as below or above DRIs. Participants were also asked about multivitamin supplement intake (yes/no).

#### 2.4.5. Physical Activity Assessment

A validated Arabic version of the Global Physical Activity Questionnaire (GPAQ) was administered during the main visit to evaluate participants’ physical activity. This tool evaluates four activity domains—occupational, transportation-related, leisure time, and sedentary behaviors—and measures frequency, duration, and intensity parameters [49]. The tool also captures comprehensive physical activity metrics by combining intensity, duration, and frequency to compute the total Metabolic Equivalent of Task (MET) minutes accumulated each week.

#### 2.4.6. Statistical Analysis

Data were analyzed using SPSS version 16.0 and presented as the mean ± standard deviation for the normal continuous variables and median (Quartile 1–Quartile 3) for non-normal variables. Categorical variables were presented in numbers and percentages. Th independent sample *t*-test and Mann–Whitney U test were used to determine differences between PSQI-P and PSQI-G quality for normal and non-normal distributed variables, respectively. Mineral levels were analyzed as continuous variables for both serum and dietary intakes (energy-adjusted). An analysis of covariance was used to determine significant differences between groups after adjusting for confounders, including age, BMI, and sex.

A logistic regression analysis was used to determine the crude and adjusted odds (OR) for poor sleep according to PSQI global scores (dependent variable) against mineral levels (independent variables). Serum minerals were modeled both as tertiles and as binary variables based on deficiency cut-offs, while dietary mineral intakes were examined as binary variables according to sex-specific DRI thresholds. Two regression models were employed: Model 1 was adjusted for age and gender, while Model 2 was further adjusted for body mass index (BMI), smoking status, physical activity, the use of medication (non-sleep aid), and supplementation intake. This analysis was performed on the entire cohort and repeated separately for male and female subjects to examine sex-related associations and for subjects <40 years and those ≥40 years to explore age-related associations. Data are expressed as ORs with corresponding 95% confidence intervals (CIs). *p* < 0.05 was considered significant. Bonferroni Corrections were applied. Stratified analyses by sex and age should be considered hypothesis-generating due to reduced statistical power and the exploratory nature of these subgroups.

## 3. Results

### 3.1. General Characteristics of Study Subjects

This study included a total of 1041 participants (Table 1), of whom 33.7% were classified as PSQI-G and 66.3% as PSQI-P, indicating a major proportion with poor sleep quality in our cohort. Compared to the PSQI-G group, the PSQI-P group was predominantly female (*p* = 0.004), exhibited greater BMIs (*p* = 0.012) and body fat percentages (*p* = 0.007), and a higher diastolic blood pressure (*p* = 0.020).

After adjusting for age, sex, and BMI, the PSQI-P group exhibited higher diastolic blood pressure (*p* = 0.005), indicating an independent association with poor sleep quality. In addition, tobacco and medication use (non-sleep aid) were more prevalent in the PSQI-P group (*p* = 0.024 for both), suggesting a covariate effect on this association. The use of supplements and physical activity scores did not differ between groups.

Overall, these findings indicate that poor sleep quality is prevalent in this cross-sectional cohort, and it is independently associated with higher diastolic blood pressure. The differences in sex distribution and adiposity appear to be confounded by demographic factors. Tobacco and medication use were also more common in PSQI-P participants only after adjustment, suggesting a covariate effect rather than an independent crude association.

### 3.2. Lower Serum Mg and Zn Levels Are Associated with Poor Sleep Quality

We compared the mean serum mineral levels between groups. Notably, the results demonstrated that the serum Mg level was lower in the PSQI-P cohort compared to the PSQI-G group (*p* = 0.007, B. Adj. = 0.036) (Table 1). Similarly, the serum Zn level was lower in the PSQI-P group (*p* = 0.019); however, the differences were attenuated after adjustment for confounders (0.232). No differences were observed between groups in serum Cu levels and in Cu/Zn ratios.

**Figure 2:**
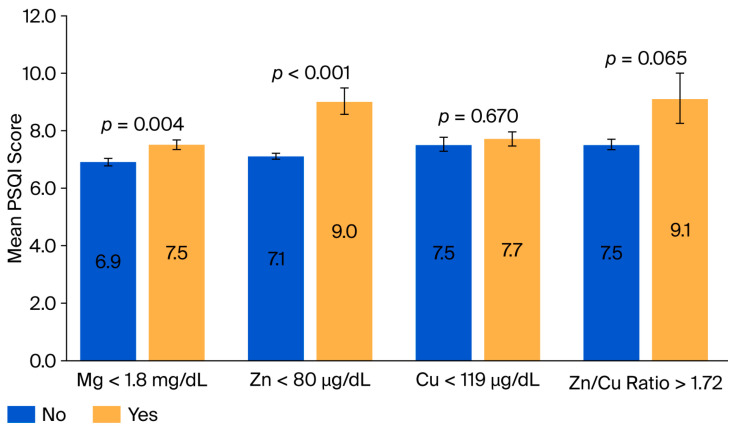
Mean Pittsburgh Sleep Quality Index (PSQI) scores grouped by dietary and serum mineral deficiency status. The mean PSQI scores were categorized by the presence of a serum mineral deficiency. Higher PSQI scores indicate poorer sleep quality. Cu, Copper; Mg, Magnesium; PSQI, Pittsburgh Sleep Quality Index; and Zn, Zinc.

When participants were classified by their serum mineral deficiency status, those with deficiencies in Zn, Mg, and Cu exhibited a trend of higher mean PSQI scores compared to those with sufficient mineral levels (Figure 2), with deficiencies in Mg and Zn showing a significant difference in the mean PSQI scores compared to sufficient states (*p* = 0.004 and *p* = 0.001, respectively).

Overall, poor sleep quality was independently associated with lower serum Mg concentrations in this cross-sectional cohort, even after adjustment for the measured covariates. The mineral deficiency analyses further revealed that Mg and Zn were associated with worse sleep quality, reinforcing the possibility of a potential role of these micronutrients in sleep regulation.

### 3.3. Highest Tertiles of Serum Zn and Cu Levels Are Associated with Lower Odds of Poor Sleep Quality

We further examined the odds of poor sleep quality across tertiles of serum mineral levels (Table 2). Compared to the lowest tertile, subjects in the highest tertile of Zn had lower odds of poor sleep quality (Zn: OR = 0.60, 95% CI 0.40–0.90; *p* = 0.040). However, Mg, Cu, and the Cu/Zn ratio did not demonstrate a tertile-related association. These findings may indicate an inverse association with poor sleep for Zn and Cu only at the highest tertiles, suggesting a threshold, rather than a linear, pattern.

### 3.4. Serum Zn and Mg Deficiencies Are Associated with Higher Odds of Poor Sleep Quality

We examined serum deficiency states for minerals of interest in relation to the odds of poor sleep quality, as measured by the PSQI (Table 3). A Zn deficiency (<80 µg/dL) was observed in 4.5% of participants and was associated with 3-fold higher odds of poor sleep quality (OR = 3.30, 95% CI = 1.40–8.00; *p* = 0.007). This association remained significant after adjustment (Model 1: OR = 3.1, CI =1.3–7.5, *p* = 0.011; Model 2: OR = 2.8, CI = 1.1–6.8, *p* = 0.024, B. Adj. = 0.043), suggesting that this relationship is independent from potential confounders, including age, sex, BMI, smoking, physical activity, hypertensive medication, and supplements.

Serum Mg deficiency (<1.8 mg/dL) was prevalent in 38.6% of participants and was associated with 1.3 times higher odds of poor sleep quality (95% CI: 1.0–1.7; *p* = 0.048). However, this association lost significance in adjusted models, suggesting this relationship may be confounded by other factors. Conversely, Cu deficiency (<119 µg/dL) was present in 48.0% of participants; however, it demonstrated no association with poor sleep quality. Similar suboptimal Cu/Zn ratios affected 4.0% of participants, and Zn deficiency demonstrated an independent association with poor sleep quality, after adjustment of measured confounders, whereas Mg and Cu deficiencies appeared to be confounded and did not exhibit independent association.

### 3.5. Sex-Related Differences in the Association Between Serum Zn and Mg Deficiency States and Odds of Poor Sleep Quality

The associations between mineral deficiencies and the odds of poor sleep quality were examined separately for female and male subjects to explore sex-related variations (Supplementary Table S1). Among males, Mg deficiency was identified in 34.8% of participants and was associated with 1.8 times higher odds of poor sleep quality (95% CI = 1.1–2.8; *p* = 0.024). This relationship remained significant in both adjusted models (Model 1: OR = 1.8, 95% CI = 1.1–2.8; *p* = 0.015; Model 2: OR= 1.7, 95% CI = 1.1–2.8; *p* = 0.024) and after the Bonferroni Correction (*p* = 0.044), suggesting an independent association with potential confounders, including age, sex, BMI, smoking, physical activity, hypertensive medication, and supplements. In contrast, Zn deficiency was uncommon (1.7%), whereas Cu deficiency affected 57.4% of male participants. A suboptimal Cu/Zn ratio was rare (1.6%), and none of these conditions were associated with the odds of poor sleep quality in any model.

Among female subjects, 6.2% were found to be Zn-deficient, and this was associated with 3.0 odds of poor sleep quality (95% CI: 1.1–7.7; *p* = 0.028). This relationship remained significant in the age-adjusted model (Model 1: OR = 3.0, 95% CI = 1.2–7.9; *p* = 0.025), with a borderline significance for further adjustments for BMI, smoking, physical activity, medication, and supplement use (Model 2: OR 2.6, 95% CI = 1.0–7.0; *p* = 0.053). In contrast, Mg deficiency was identified in 40.9% of female participants, Cu deficiency was identified in 43.6%, and a suboptimal Cu/Zn ratio was identified in 5.1%, yet none of these conditions was associated with the odds of poor sleep quality in any model. In summary, these findings from this cross-sectional cohort suggest sex-related differences, with Mg deficiency being independently associated with higher odds of poor sleep quality in males and serum Zn deficiency demonstrating a less robust association with poor sleep in females.

### 3.6. Age-Related Differences in the Association Between Serum Zn and Mg Deficiency States and Higher Odds of Poor Sleep Quality

We examined mineral deficiencies and their association with poor sleep quality, as measured by the PSQI, among participants stratified by age groups (Supplementary Table S2). For subjects <40 years old, Mg deficiency was observed in 39.4% and was associated with 1.5 odds of poor sleep quality (95% CI: 1.1–2.0; *p* = 0.021), indicating a significant association with poor sleep quality. In the age-adjusted model, the OR remained significant (Model 1: OR = 1.4, 95% CI = 1.0–2.0; *p* = 0.033). However, further adjustment for BMI, smoking, physical activity, medication, and supplement use attenuated this association (OR = 1.4, 95% CI = 1.0–1.9; *p* = 0.071). Lastly, 52.4% of participants had Cu deficiency, 4.3% exhibited Zn deficiency, and 3.3% had a suboptimal Cu/Zn ratio. However, no associations with the odds of poor sleep quality were identified.

For subjects ≥40 years old, Zn deficiency was present in 5.1%, and this was associated with marked (17.47) odds of poor sleep quality (95% CI: 1.0–303.1; *p* = 0.047). However, this association was not further explored in the adjusted models due to the small group size. In addition, 39.8% of subjects over 40 years old had a Cu deficiency, and 36.2% had a Mg deficiency. However, these deficiencies were not associated with odds of poor sleep quality across all models. Together, our findings highlight potential age-related differences in the association between mineral deficiencies and sleep quality. However, due to the small number of deficient cases in this age group, these results should be interpreted with caution and require further exploration.

### 3.7. Lower Dietary Mg Intake Is Associated with Odds of Poor Sleep Quality

Using three nonconsecutive 24 h dietary recalls, we assessed energy-adjusted dietary mineral intakes (Table 1). Compared with the PSQI-G group, those with PSQI-P had a lower energy-adjusted dietary Mg intake (*p =* 0.015), and this difference remained significant after adjustment for age, sex, and BMI (*p =* 0.005). No significant differences were observed for dietary Zn or Cu intake in the crude or adjusted analyses.

When evaluating the mineral dietary intake in relation to Dietary Reference Intake (DRI) cut-offs (Table 3), 94.2% of participants consumed Mg concentrations below the DRI level, and this was associated with higher odds of poor sleep (crude OR = 2.2, 95% CI: 1.3–3.7, *p =* 0.003); this association persisted after adjustment for age and sex (Model 1 OR = 1.9, *p =* 0.025) and after full adjustment for BMI, smoking, physical activity, medication, and supplement use (Model 2 OR = 1.8, *p = 0.04*). However, the significance was lost after Bonferroni Correction (*p* = 0.120). In contrast, suboptimal dietary Zn and Cu intakes were present in 74.4% and 38.2% of participants, respectively. Both were not associated with poor sleep quality. Overall, these findings show that a lower dietary Mg intake was associated with poorer sleep quality after adjustment for the measured covariates. This demonstrates that dietary Mg intake has an independent association with poor sleep quality in our cohort. In contrast, suboptimal dietary Zn and Cu intakes, despite being common in our cohort, were not associated with sleep quality.

## 4. Discussion

In this cross-sectional study, we examined the associations between serum and dietary intake levels of Mg, Zn, and Cu with sleep quality, as measured by the PSQI, in a cohort of Saudi adults. Our main findings highlight a high prevalence of poor sleep quality in our cohort and reveal the following: (1) Mg is a factor in poor sleep quality, with lower serum levels, a lower energy-adjusted dietary intake, and an intake below DRIs each exhibiting independent associations with poor sleep, after adjustment for the measured covariates. (2) Serum Zn deficiency, but not dietary intake, is independently associated with poor sleep quality. (3) We observed sex-specific patterns; in males, serum Mg deficiency was independently associated with higher odds of poor sleep. (4) Serum or dietary intake levels of Cu demonstrated no association with sleep quality. However, that the findings may not extend to rural populations, older adults with multimorbidity, or clinical settings in which both sleep disturbances and mineral deficiencies are more prevalent.

Poor sleep quality was prevalent in our cohort, affecting nearly two-thirds of participants. This is consistent with other reports of poor sleep quality in Saudi adults of different groups, ranging between 60 and 80% [50,51,52,53]. A higher rate of poor sleep quality in female subjects was reported in [52], which is in agreement with our findings that reveal that poor sleepers were predominantly females. Poor sleepers also exhibited higher adiposity (as BMI and fat %) and diastolic blood pressure. The association between poor sleep and diastolic blood pressure persisted after adjusting for age, sex, and BMI, suggesting a potential direct link between sleep disturbances and vascular health. The higher prevalence of tobacco and medication use (other than sleep aids) among poor sleepers observed only after adjustment supports the covariate effect, potentially reflecting behavioral and metabolic factors or comorbidities that cluster with poor sleep.

Mg is thought to influence sleep through multiple neurobiological pathways, including the modulation of GABA and the glutamate NMDA receptors; participation in melatonin and serotonin synthesis; regulation of cortisol and stress axis signaling; and modulation of inflammation, oxidative stress, the excitability of neurons, and muscle relaxation [25]. Growing evidence indicates a favorable effect of Mg on sleep quality, with several studies reporting an inverse association between serum/plasma Mg levels and sleep quality parameters in adults [24,29,31,32]. Our findings align with this research and demonstrate an association between Mg status and sleep quality across both serum and dietary indices. Lower serum Mg levels were associated with poor sleep independent of age, sex, and BMI. However, the tertile analysis across the observed range (0.8–2.3 mg/dL) did not reveal a linear dose–response pattern. This indicates that lifestyle and metabolic factors may partly mediate this relationship. The attenuation may also reflect the limited utility of binary deficiency classifications in relation to sleep outcomes. It may also suggest a threshold effect in which sleep quality deteriorates only when Mg levels fall below a critical range relevant to sleep physiology, with no progressive change within normal levels. Taken together, these findings support an overall role of Mg in sleep regulation and suggest that maintaining Mg levels above a minimal physiological threshold may be more relevant for sleep health. The fact that nearly 40% of participants had a serum Mg deficiency underscores its high prevalence and warrants further inspection, especially as a modifiable factor related to sleep health.

Sex- and age-stratified analyses suggest heterogeneity in the association of Mg with sleep quality. Mg deficiency was more prevalent in males (40.9%) than females (34.8%) and was associated with nearly 1.8-fold higher odds of poor sleep quality, even after adjusting for age, BMI, smoking, physical activity, medication, and supplement use. This supports an association of Mg on sleep quality in males, potentially reflecting sex-specific biological differences, including the hormonal milieu, adiposity, and body composition. An age-specific effect of Mg has been previously reported, where higher serum levels appeared to be protective against sleep disturbances primarily in middle-aged adults (30–59 years) [29]. Our findings are in agreement and show that in adults (<40 years), Mg deficiency was associated with 1.5-fold higher odds of poor sleep quality; however, this was attenuated to a non-significant status after adjustment for confounders. Therefore, the age-related association in younger individuals appears to be mediated by confounding lifestyle and/or metabolic factors that warrant further assessment. These data are exploratory and require further studies.

In line with the serum findings, dietary Mg intake was found have an independent association with poor sleep quality after adjustment for the measured covariates. In our study, poor sleepers reported a significantly lower energy-adjusted Mg intake in both the crude and adjusted models, indicating that a lower Mg intake is consistently linked with poorer sleep. Moreover, when intake was evaluated against DRI cut-offs, 94% of participants consumed less than the recommended level, and such a suboptimal intake was associated with higher odds of poor sleep, with the association remaining significant after adjusting for the measured confounders. These findings align with previous work showing that lower dietary Mg intake is associated with poor sleep quality in adults [54]. It also agrees with longitudinal data indicating that participants in the highest quartile of Mg intake had lower odds of short sleep durations (<7 h) compared with those in the lowest quartile, despite only borderline associations with self-rated sleep quality (rated as very good to very poor) [36]. Collectively, our findings demonstrate that a low dietary Mg intake is associated with poor sleep quality and highlight Mg insufficiency as a common, potentially modifiable dietary issue in this cohort. Its worthy to note that dietary intake misclassification and systematic error may influence both prevalence estimates and associations.

Zn may influence sleep quality through several mechanisms, including by modulating GABA and NMDA receptor activity, supporting inhibitory tones and limiting glutamatergic hyperexcitability, cofactors in the tryptophan–serotonin–melatonin pathway, and melatonin signaling [26]. Findings from previous studies demonstrated that the serum/plasma Zn concentration is inversely associated with the risk of poor sleep quality measured by the PSQI or self-reported sleep disorders in adults [27,28,29,30,33]. Our data are in agreement and demonstrate an inverse association between serum Zn and poor sleep quality across continuous, binary, and tertile indices. Serum Zn concentration was examined by tertiles across the observed range (80–166 µg/dL), participants in the highest Zn tertile had a 40% reduction in the odds of poor sleep than those in the lowest tertile, suggesting a graded inverse association of Zn within the higher physiological range. Moreover, when the serum Zn concentration was classified using a binary deficiency cut-off, a Zn deficiency, despite being uncommon (4.5%), was associated with markedly higher PSQI scores and a 3-fold increase in the odds of poor sleep—an association that remained significant after multivariable adjustments, supporting an independent role of Zn deficiency in impairing sleep physiology and highlighting a threshold effect, consistent with prior reports suggesting that the Zn–sleep relationship may depend on optimal ranges or threshold patterns [28,30,54]. Collectively, these findings underscore the potential role of the Zn status in sleep regulation. This indicates that while serum Zn levels demonstrate a modest association with sleep quality, higher physiological Zn concentrations appear to be protective, and clinically, Zn deficiency represents a distinct, state associated with for poor sleep. This also suggests that Zn supports sleep quality through both a graded, dose–response inverse association within the normal physiological range and a deficiency threshold beyond which sleep quality is substantially deteriorated. It is important to note here that the data were exploratory, driven by small numbers, and are not suitable for strong mechanistic interpretations.

We observed potential sex- and age-specific differences in the relationship between serum Zn status and sleep quality. Among female subjects, Zn deficiency was more prevalent (6.2%) than in males (1.7%) and showed 3-fold higher odds of poor sleep (lost significance after further adjustments). Together, these data suggest that metabolic and lifestyle factors may mediate or confound the observed sex-specific association of Zn with sleep quality. An age-related pattern was previously reported in a follow-up study examining Zn–sleep associations at different ages from preschool age to early adolescence [55]. In our cohort, Zn deficiency was similarly prevalent across age groups (5.1% in adults ≥40 years vs. 4.3% in those <40 years), but among older adults (≥40 years). However, due to the small sample, this association was not further examined in multivariable models. The observed age-related vulnerability may reflect coexisting comorbidities and/or age-related physiological changes in Zn absorption and utilization. Notably, the BMI was higher in older adults (29.9 vs. 25.8 kg/m^2^, *p* < 0.001), which could partially confound or mediate the association. Altogether, the observed sex-related differences in Zn’s association with sleep are not independent and may be confounded by metabolic and lifestyle factors, and the age-related patterns, although pronounced in crude models, remain exploratory and require confirmation in larger studies with adequately powered, fully adjusted analyses.

Previous studies in female subjects have reported inverse associations between dietary Zn intake and poor sleep [27,56]. In our cohort, poor sleepers reported lower energy-adjusted Zn intakes compared to good sleepers in crude analyses. However, this association lost significance after adjusting for age, sex, and BMI, indicating that the association is explained by these factors. Although 28% of participants had suboptimal dietary Zn intake, such exposure did not are associated with poor sleep in any model. Collectively, our data indicates that dietary Zn is a weak proxy to predict physiological effects on sleep. This also raises the possibility that the DRI cut-off may not represent a physiologically meaningful threshold for sleep-related outcomes, motivating research to define functional, sleep-relevant targets rather than deficiency prevention levels.

In previous research, serum Cu level was reported to have a negative association with sleep quality in male and female adults [30,34]; however, the findings are not consistent. Copper may influence sleep quality by serving as a cofactor for enzymes involved in neurotransmitter synthesis that regulate sleep–wake cycles and by modulating oxidative stress and inflammatory pathways, which are linked to sleep disturbances and circadian dysregulation [53,54]. One study found no significant relationship between serum Cu levels and sleep disorders [28], whereas another study reported a U-shaped association between Cu and sleep disorder risks in both males and females and in older adults (>60 years), suggesting that both low and high Cu levels may confer risk [35]. In our cohort, mean serum Cu levels did not differ between good and poor sleepers, and despite 48% of participants being classified as Cu-deficient, this status did not predict PSQI scores in any model. Notably, within the observed range (86–150 µg/dL) individuals in the highest Cu tertile exhibited 30% lower odds of poor sleep compared with the lowest tertile, indicating a modest inverse association at higher physiological levels. This pattern is more consistent with a threshold–effect relationship, which warrants further investigation. The absence of sex- or age-specific trends implies that any influence of Cu on sleep, if present, may act through common metabolic pathways rather than subgroup-specific mechanisms. Overall, the Cu status appears unlikely as a major independent determinant of sleep quality. In the current analysis, energy-adjusted dietary Cu levels were lower in poor sleepers (*p* = 0.059) in crude analyses, but this difference disappeared after adjustment, and a suboptimal Cu intake (below DRI) was not associated with poor sleep in any model, suggesting no meaningful link between dietary Cu and sleep quality.

The serum Cu/Zn ratio is interpreted as a marker of the trace element balance and oxidative stress and inflammatory status [55,57]. Multiple studies reported that a lower serum Cu/Zn ratio (or higher Zn/Cu ratio) is associated with better sleep outcomes [28,29,30,34]. In our cohort, the Cu/Zn ratio exhibited no association with sleep quality. Participants with higher Cu/Zn ratios (>1.72) exhibited a trend of higher PSQI scores (*p* = 0.077) in the crude and age-adjusted models, but these associations attenuated after full adjustment for confounders, suggesting a role in the observed association. However, the small number of subjects with suboptimal Cu/Zn ratios (4.0%) likely reduced the power to detect any significant differences, which may explain the borderline trends. Our analysis is limited by the cross-sectional design, which precludes causal inference between mineral status and sleep outcomes. Dietary intake data were assessed using the self-reported 24 h food recall, which is subject to recall bias and measurement errors and is unlikely to capture habitual intake. Although repeated recalls were used to improve the estimation of habitual intake and dietary intakes demonstrated a good correlation with serum biomarkers, the possibility of misclassification of nutrient intake cannot be excluded. DRI non-adherence rates, particularly for Mg, may overestimate true inadequacy due to systematic recall underreporting. Thus, the high prevalence of participants below the DRI for magnesium should therefore be interpreted cautiously. Sleep recall may also be subject to bias. This study involved multiple statistical comparisons across different analytical approaches. While Bonferroni Corrections were applied, some findings may represent type I errors. Additionally, stratified analyses by sex and age had reduced power and should be considered hypothesis-generating. Estimates with extremely wide confidence intervals reflect small subgroup sizes and warrant verification in larger samples. Additionally, our sample represents a convenience cohort of relatively healthy adults rather than a probabilistic or nationally representative Saudi population. As such, our findings may not be extended to rural populations, older adults with multimorbidity, or clinical populations where both sleep disturbances and mineral deficiencies are more prevalent. Despite these limitations, to our knowledge, this study is the first cross-sectional convenience study conducted among an apparently healthy sample from Riyadh. This study provides a parallel examination of both biochemical and dietary levels of Zn, Mg, and Cu and their combined ratios in relation to sleep quality. It also examines multiple mineral metrics, including continuous levels, deficiency thresholds, and combined indices. Furthermore, the sex-stratified analyses provide novel insights into possible heterogeneity in the effect of mineral deficiencies on sleep-related outcomes.

## 5. Conclusions

In conclusion, this cross-sectional study conducted with an apparently healthy adult sample provided a concurrent examination of serum and dietary intake levels of Mg, Zn, and Cu in relation to sleep quality. Our findings highlight that poor sleep was highly prevalent in our cohort of Saudi adults and is generally associated with elevated diastolic blood pressure. Sleep quality was modestly associated with mineral status, particularly Mg and Zn. Both serum and dietary Mg statuses were associated with sleep quality after adjustment for the measured covariates. Lower serum Mg levels and its deficiency (<1.8 mg/dL) were associated with higher odds of poor sleep. In addition, dietary Mg intake (energy-adjusted) and suboptimal intake (below DRI) were associated with sleep quality even after adjustment. Serum Zn levels, not dietary levels, were associated with sleep quality, with higher physiological Zn levels conferring modest protection and clinical Zn deficiency (<80 µg/dL) representing a state associated with poor sleep. Both Mg and Zn demonstrated apparent sex- and age-related patterns in crude analyses; however, these subgroup findings should be interpreted cautiously and considered hypothesis-generating only, given attenuation after adjustment, sensitivity to multiple testing, and limited precision. Thus, only Mg deficiency was identified as an independent factor for poor sleep quality in males after adjustment for measured confounders. Cu indices exhibited limited associations with sleep quality. While a modest protective association was observed at the highest serum tertile, this pattern was not confirmed by serum deficiency or dietary analyses. In contrast, the Cu/Zn ratio demonstrated no association with sleep quality.

The data suggest that Mg primarily influences sleep through a threshold-based mechanism, wherein sleep quality deteriorates once the serum Mg level falls below a critical range, without progressive benefits at higher levels. In contrast, Cu exhibits a graded inverse association across the normal physiological range, and Zn appears to act through two modes—a graded protective effect and a deficiency threshold. Importantly, most subgroup, deficiency-based, and stratified findings should be considered hypothesis-generating, as several associations attenuated after full adjustment were sensitive to multiple testing and were accompanied by wide confidence intervals. Future research should include longitudinal and interventional studies to establish causality and to define clinically meaningful mineral targets for sleep health beyond simple deficiency thresholds before any public health or clinical recommendations can be made.

## Figures and Tables

**Table 1:** Baseline characteristics of participants grouped by sleep quality (PSQI), highlighting key differences in magnesium and zinc status.

Parameters	PSQI-G (PSQI ≤ 5)	PSQI-P (PSQI > 5)	*p*-Value ^⁂^	*p*-Value (B. Adj.)
N	351	690		
Age (years)	32.7 ± 12.8	31.3 ± 11.9	0.082	--
BMI (kg/m^2^)	26.1 ± 5.8	27.2 ± 6.3	0.012	--
Male/Female	155/196	242/448	0.004	--
Fat (%)	33.6 ± 10.5	35.4 ± 10.6	0.007	0.237
SBP (mmHg)	112.4 ± 15.3	111.1 ± 15.9	0.216	0.597
DBP (mmHg)	73.6 ± 10.5	75.3 ± 10.9	0.020	0.005 *
Serum Mg (mg/dL)	1.8 ± 0.5	1.7 ± 0.4	0.007	0.015 * (0.036)
Serum Zn (µg/dL)	116.4 ± 26.4	112.2 ± 26.0	0.019	0.058 (0.232)
Serum Cu (µg/dL)	117.1 ± 18.9	116.7 ± 18.4	0.835	0.311 (1.00)
Serum Cu/Zn Ratio	1.2 ± 0.2	1.2 ± 0.3	0.154	0.730 (1.00)
Dietary Mg (per 1000 Kcal)	108.7 (89.3–136.5)	104.4 (84.4–129.3)	0.015	0.005 * (0.015)
Dietary Zn (per 1000 Kcal)	3.8 (3.0–4.7)	3.6 (2.9–4.5)	0.066	0.716 (1.00)
Dietary Cu (per 1000 Kcal)	0.5 (0.4–0.7)	0.5 (0.4–0.6)	0.059	0.123 (0.369)
GPAQ Score	199 (56.7)	366 (53.0)	0.264	0.477
Smoking (Yes, %)	41 (11.7)	105 (15.2)	0.120	0.024
Prescription Medication Use (Not Sleep Aid) (Yes, %)	17 (4.8)	48 (7.0)	0.183	0.024
Multivitamin Supplement Intake (Yes, %)	81 (23.1)	181 (26.2)	0.267	0.195

Note: Data are presented as Mean ± SD for normal variables and median (Quartile 1–Quartile 3) for non-normal variables; *p* < 0.05 is considered significant. ⁂ Indicates *p*-values adjusted for age, sex, and BMI; * indicates statistically significant associations. Energy-adjusted dietary mineral intake was derived from three nonconsecutive 24 h dietary recalls. BMI, Body Mass Index; Cu, Copper; DBP, Diastolic Blood Pressure; GPAQ, Global Physical Activity Questionnaire; Mg, Magnesium; PSQI, Pittsburgh Sleep Quality Index; SBP, Systolic Blood Pressure; PSQI-G, Good Sleep Quality; PSQI-P, Poor Sleep Quality; and Zn, Zinc.; Bonferroni Correction Applied (B. Adj.): Separate corrections were performed for the two families of tests. Serum Nutrient Variables (4 tests): *p*-value threshold = 0.05/4 = 0.0125. Dietary Nutrient Variables (3 tests): *p*-value threshold = 0.05/3 = 0.0167. This table provides descriptive characteristics. Among the mineral measures, differences were observed primarily for magnesium serum and dietary levels, whereas other minerals did not differ between sleep quality groups.

**Table 2:** Tertile-specific odds of poor sleep quality by serum mineral levels.

Mineral/Index		Tertile/Quantile	Tertile/Quantile (B. Adj.)	Tertile/Quantile (B. Adj.)
Mg	Mean Serum Level (mg/dL)	1.2 ± 0.4	1.9 ± 0.1	2.1 ± 0.2
	OR for PSQI-P (95% CI)	--	1.2 (0.7–2.0)	0.7 (0.4–1.2)
	*p*-value (B. Adj.)	--	0.569 (1.000)	0.226 (1.000)
Zn	Mean Serum Level (µg/dL)	90.6 ± 10.5	109.1 ± 4.4	141.8 ± 24.0
	OR for PSQI-P (95% CI)	--	1.0 (0.7–1.4)	0.6 (0.4–0.9)
	*p*-Value (B. Adj.)	--	0.901 (1.000)	0.006 (0.040)
Cu	Mean Serum Level (µg/dL)	95.1 ± 9.3	119.5 ± 6.0	137.1 ± 5.9
	OR for PSQI-P (95% CI)	--	0.9 (0.6–1.3)	0.7 (0.5–0.8)
	*p*-Value (B. Adj.)	--	0.554 (1.000)	0.036 (0.280)
Cu/Zn ratio	Mean Serum Level	0.9 ± 0.1	1.2 ± 0.1	1.5 ± 0.2
	OR for PSQI-P (95% CI)	--	1.3 (0.8–2.3)	1.3 (0.7–2.2)
	*p*-Value (B. Adj.)	--	0.315 (1.000)	0.343 (1.000)

Note: Data are presented as Mean ± SD for normal variables and median (Quartile 1–Quartile 3) for non-normal variables; *p* < 0.05 is considered significant. Cu, Copper; Mg, Magnesium; PSQI, Pittsburgh Sleep Quality Index; PSQI-G, Good Sleep Quality; PSQI-P, Poor Sleep Quality; and Zn, Zinc. Bonferroni Correction Applied (B. Adj.): Serum nutrient variables (4 tests): *p*-value threshold = 0.05/4 = 0.0125. Copper and Cu/Zn ratio tertile analyses are exploratory and revealed no consistent independent associations.

**Table 3:** Mineral serum deficiencies or suboptimal dietary intake levels and odds of poor sleep quality measured by Pittsburgh Sleep Quality Index (PSQI) score.

Mineral/Index	Deficient	Crude		Model 1		Model 2	
	N (%)	OR (95% CI)	*p*-Value (B. Adj.)	OR (95% CI)	*p*-Value (B. Adj.)	OR (95% CI)	*p*-Value (B. Adj.)
Mg deficiency (<1.8 mg/dL)	360 (38.6)	1.3 (1.0–1.7)	0.048 * (0.065)	1.3 (1.0–1.7)	0.081 (0.324)	1.2 (0.9–1.6)	0.147 (0.588)
Zn deficiency (<80 µg/dL)	42 (4.5)	3.3 (1.4–8.0)	0.007 * (0.028)	3.1 (1.3–7.5)	0.011 * (0.044)	2.8 (1.1–6.8)	0.024 * (0.043)
Cu deficiency (<119 µg/dL)	182 (48.0)	1.0 (0.7–1.6)	0.868 (1.000)	1.0 (0.7–1.6)	0.892 (1.000)	1.2 (0.7–1.9)	0.461 (1.000)
Cu/Zn ratio (>1.72 µg/dL)	15 (4.0)	6.3 (0.8–48.5)	0.077 (0.308)	6.3 (0.8–49.2)	0.078 (0.312)	5.0 (0.6–40.3)	0.131 (0.524)
Dietary Mg < DRI	981 (94.2)	2.2 (1.3–3.7)	0.003 (0.009)	1.9 (1.1–3.2)	0.025 (0.075)	1.8 (1.0–3.1)	0.04 (0.120)
Dietary Zn < DRI	774 (74.4)	1.2 (0.9–1.6)	0.178 (0.534)	1.0 (0.7–1.4)	0.914 (1.000)	1.1 (0.8–1.5)	0.67 (1.000)
Dietary Cu < DRI	398 (38.2)	1.1 (0.8–1.4)	0.57 (1.000)	1.0 (0.8–1.3)	0.911 (1.000)	1.0 (0.8–1.3)	0.57 (1.000)

Note: Data are presented as odds ratio (95%CI). Dietary mineral intake was derived from three energy-adjusted nonconsecutive 24 h dietary recalls. Model 1, adjusted for age and sex. Model 2 adjusted for age, sex, BMI, smoking, physical activity, hypertensive medication, and supplements. * Indicates statistically significant associations. Cu, Copper; DRI, Dietary Reference Intake; Mg, Magnesium; OR, Odds Ratio; and Zn, Zinc; Bonferroni Correction Applied: Separate corrections were performed for the two families of tests. Serum Variables (4 tests): *p*-value threshold = 0.05/4 = 0.0125. Dietary Variables (3 tests): *p*-value threshold = 0.05/3 = 0.0167. Only serum zinc deficiency and dietary magnesium deficiency in the crude model remain significant after their respective corrections.

## Data Availability

The data presented in this study are available from the corresponding author upon request due to ethical consideration and privacy proception requirements.

## Supplementary Materials

The following supporting information can be downloaded at: https://www.mdpi.com/article/10.3390/nu18010114/s1, Table S1: Exploratory sex-stratified associations between mineral deficiencies and poor sleep quality. Table S2: Exploratory age-stratified associations between mineral deficiencies and poor sleep quality.

## Abbreviations

The following abbreviations are used in this manuscript: BIA, Bioelectrical Impedance Analysis; BMI, Body Mass Index; CI, Confidence Interval; Cu, Copper; Cu/Zn Ratio, Copper-to-Zinc Ratio; DRI, Dietary Reference Intake; GABA, Gamma-Aminobutyric Acid; GPAQ, Global Physical Activity Questionnaire; Kcal, Kilocalories; MENA, Middle East and North Africa; MET, Metabolic Equivalent of Task; Mg, Magnesium; NMDA, N-methyl-D-aspartate; OR, Odds Ratio; PSQI, Pittsburgh Sleep Quality Index; PSQI-G, Good Sleep Quality Group; PSQI-P, Poor Sleep Quality Group; SD, Standard Deviation; Zn, Zinc; Zn/Cu Ratio, Zinc-to-Copper Ratio.
