# Supplementary material for: Magnesium and Zinc Are Associated with Sleep Quality in Saudi Adults: Evidence from a Cross-Sectional Study
Source: Nutrients. 2025 Dec 29;18(1):114. doi: 10.3390/nu18010114 (PMC12787342; doi:10.3390/nu18010114)
Supplement: Supplementary file 1 [file nutrients-18-00114-s001.zip › nutrients-4052201-supplementary.pdf]

Supplementary Table S1: Exploratory sex-stratified associations between mineral deficiencies and poor sleep quality

Mineral/Index		Deficient	Crude		Model 1		Model 2	
		N (%)	OR (95%CI)	*p*-value	OR (95%CI)	*p*-value	OR (95%CI)	*p*-value
Mg deficiency (< 1.8 mg/dL)	Female	237 (40.9)	1.1 (0.7 – 1.5)	0.721 (1.000)	1.1 (0.7 – 1.5)	0.773 (1.000)	1.0 (0.7 – 1.4)	0.968 (1.000)
	Male	123 (34.8)	1.8 (1.1 – 2.8)	0.014 (0.056)	1.8 (1.1 – 2.8)	0.015 (0.060)	1.7 (1.1 – 2.8)	0.024 (0.044)
Zn deficiency (< 80 µg/dL)	Female	36 (6.2)	3.0 (1.1 – 7.7)	0.028 (0.112)	3.0 (1.2 – 7.9)	0.025 (0.100)	2.6 (1.0 – 7.0)	0.053 (0.212)
	Male	6 (1.7)	3.5 (0.4 – 30.0)	0.259 (1.000)	3.5 (0.4 – 30.5)	0.254 (1.000)	4.4 (0.5 – 39.3)	0.186 (0.744)
Cu deficiency (< 119 µg/dL)	Female	112 (43.6)	1.2 (0.7 – 2.1)	0.479 (1.000)	1.1 (0.6 – 2.0)	0.635 (1.000)	1.2 (0.7 – 2.2)	0.517 (1.000)
	Male	70 (57.4)	0.9 (0.4 – 1.9)	0.774 (1.000)	1.0 (0.4 – 1.9)	0.783 (1.000)	1.1 (0.5 – 2.7)	0.769 (1.000)
Cu/Zn ratio (> 1.72 µg/d)	Female	13 (5.1)	4.7 (0.6 – 37.1)	0.139 (0.556)	4.9 (0.6 – 38.6)	0.131 (0.524)	3.0 (0.4 – 24.9)	0.303 (1.000)
	Male	2 (1.6)	2.9 (0.1 – 61.9)	0.494 (1.000)	--	--	--	--

Note: Data presented as Odds Ratio (95%CI); Model 1, adjusted for age; Model 2 adjusted for age, BMI, smoking, physical activity, hypertensive medication and supplements.; * indicated statistically significant association; Mg, Magnesium; Zn, Zinc; Cu, Copper. Bonferroni Correction Applied: Correction was performed separately for males and females. Each sex-specific analysis constitutes a family of 4 hypotheses (Mg, Zn, Cu, Cu/Zn). The significance threshold for each family is 0.05 / 4 = 0.0125; Only Mg Deficiency among male remined border significant. Others, no association remains statistically significant after applying the sex-specific Bonferroni correction (i.e., no adjusted p-value < 0.0125). Results nominally significant at p < 0.05 in the raw analysis should be interpreted as exploratory.

Supplementary Table S2: Exploratory age-stratified associations between mineral deficiencies and poor sleep quality

Mineral/Index		Deficient	Crude		Model 1		Model 2	
		N (%)	OR (95%CI)	*p*-value	OR (95%CI)	*p*-value	OR (95%CI)	*p*-value
Mg deficiency (< 1.8 mg/dL)	< 40 Years	275 (39.4)	1.5 (1.1 – 2.0)	0.021 (0.084)	1.4 (1.0 – 2.0)	0.033 (0.132)	1.4 (1.0 – 1.9)	0.071 (0.284)
	≥ 40 Years	85 (36.2)	1.0 (0.6 – 1.7)	0.92 (1.000)	1.4 (1.0 – 2.0)	0.897 (1.000)	1.0 (0.6 – 1.7)	0.944 (1.000)
Zn deficiency (< 80 µg/dL)	< 40 Years	30 (4.3)	2.0 (0.8 – 5.0)	0.126 (0.504)	1.6 (0.5 – 6.0)	0.443 (1.000)	1.7 (0.7 – 4.2)	0.271 (1.000)
	≥ 40 Years	12 (5.1)	17.47 (1.0 -303.1)	0.047 (0.188)	--	--	--	--
Cu deficiency (< 119 µg/dL)	< 40 Years	129 (52.4)	1.0 (0.6 – 1.8)	0.969 (1.000)	1.1 (0.6 – 1.8)	0.862 (1.000)	1.3 (0.7 – 2.5)	0.342 (1.000)
	≥ 40 Years	53 (39.8)	1.0 (0.5 – 2.0)	0.92 (1.000)	1.0 (0.5 – 2.2)	0.911 (1.000)	1.1 (0.5 - 2.3)	0.874 (1.000)
Cu/Zn ratio (> 1.72 µg/d)	< 40 Years	8 (3.3)	6.7 (0.4 -117.5)	0.194 (0.776)	--	--	--	--
	≥ 40 Years	7 (5.3)	1.0 (0.6 – 1.7)	0.92 (1.000)	1.4 (1.0 – 2.0)	0.897 (1.000)	1.0 (0.6 – 1.7)	0.944 (1.000)

Note: Data presented as Odds Ratio (95%CI); Model 1, adjusted for age; Model 2 adjusted for age, BMI, smoking, physical activity, hypertensive medication and supplements.; * indicated statistically significant association; Mg, Magnesium; Zn, Zinc; Cu, Copper. Bonferroni Correction Applied: Correction was performed separately for age groups. Each age-specific analysis constitutes a family of 4 hypotheses (Mg, Zn, Cu, Cu/Zn). The significance threshold for each family is 0.05 / 4 = 0.0125; No association remains statistically significant after applying the age-specific Bonferroni correction (i.e., no adjusted p-value < 0.0125). Results nominally significant at p < 0.05 in the raw analysis should be interpreted as exploratory.
